# Impact of dopant-induced band tails on optical spectra, charge carrier transport, and dynamics in single-crystal CdTe

**DOI:** 10.1038/s41598-022-16994-7

**Published:** 2022-07-27

**Authors:** Patrik Ščajev, Algirdas Mekys, Liudvikas Subačius, Sandra Stanionytė, Darius Kuciauskas, Kelvin G. Lynn, Santosh K. Swain

**Affiliations:** 1 Institute of Photonics and Nanotechnology, Faculty of Physics, Vilnius University, Saulėtekio Ave. 3, 10257 Vilnius, Lithuania; 2 Optoelectronics Department, Center for Physical Sciences and Technology, Saulėtekio Ave. 3, 10257 Vilnius, Lithuania; 3 National Renewable Energy Laboratory, 15013 Denver West Parkway, Golden, CO 80401 USA; 4 Center for Materials Research, Washington State University, Pullman, WA 99164 USA

**Keywords:** Solar energy, Solar cells

## Abstract

Cadmium telluride (CdTe) semiconductors are used in thin-film photovoltaics, detectors, and other optoelectronic applications. For all technologies, higher efficiency and sensitivity are achieved with reduced charge carrier recombination. In this study, we use state-of-the-art CdTe single crystals and electro-optical measurements to develop a detailed understanding of recombination rate dependence on excitation and temperature in CdTe. We study recombination and carrier dynamics in high-resistivity (undoped) and arsenic (As)-doped CdTe by employing absorption, the Hall effect, time-resolved photoluminescence, and pump-probe in the 80–600 K temperature range. We report extraordinarily long lifetimes (30 µs) at low temperatures in bulk undoped CdTe. Temperature dependencies of carrier density and mobility reveal ionization of the main acceptors and donors as well as dominant scattering by ionized impurities. We also distinguish different recombination defects. In particular, shallow As_Te_ and deep V_Cd_−As_Cd_ acceptors were responsible for p-type conductivity. AX donors were responsible for electron capture, while nonradiative recombination centers (V_Cd_−As_Te_, As_2_ precipitates), and native defects (V_Cd_−Te_Cd_) were found to be dominant in p-type and n-type CdTe, respectively. Bimolecular and surface recombination rate temperature dependencies were also revealed, with bimolecular coefficient T^−3/2^ temperature dependence and 170 meV effective surface barrier, leading to an increase in surface recombination velocity at high temperatures and excitations. The results of this study allowed us to conclude that enhanced crucible rotation growth of As-doped CdTe is advantageous to As activation, leading to longer lifetimes and larger mobilities and open-circuit voltages due to lower absorption and trapping.

Owing to its optoelectronic and chemical properties, cadmium telluride (CdTe) is an ideal material for thin-film polycrystalline solar cells. The current power conversion efficiency record is 22.1%, and simulations indicate pathways to 25% efficient CdTe solar cells^1,2^. To achieve such efficiencies, modeling suggests that absorber doping needs to reach 10^16^ cm^−3^, carrier lifetime needs to exceed 100 ns, and interface recombination velocity needs to be reduced below 1000 cm/s^1^. Doping CdTe is a significant challenge. Doping with Cu is limited by compensation^3^, and Cu-doped absorbers can be metastable^4^. Recent progress with Sb, P, and As dopants suggests that ≥ 10^16^ cm^−3^ doping could be achieved^5–8^. Because As doping can be used in large-scale photovoltaics manufacturing, it has been the most actively studied^9,10^. However, As doping can lead to carrier lifetime degradation^11^. Recombination in polycrystalline absorbers depends on grain boundaries, dopant aggregation at interfaces, and other difficult-to-control variables. For solar cells, polycrystalline absorbers have a graded (depth-dependent) bandgap, different passivation at the front and back interfaces, and space charge fields. It is difficult to determine properties such as minority carrier lifetime and mobility in such complex absorbers^12^. Therefore, although first principles analysis has identified defects in As-doped CdTe^13^, such predictions largely have not been verified experimentally^14^.

Single crystals are ideal model systems for understanding doping, recombination, and transport, and substantial effort has been applied to grow As-doped single crystals and incorporate them into solar cells^5,15^. Nagaoka et al. developed CdTe single-crystal growth using the traveling heater method (THM)^16^ and reported mobilities and carrier lifetimes, and they achieved very high dopant activation in THM single-crystal CdTe^17,18^. THM growth is slow, and it is only possible for small crystals. In contrast, the vertical Bridgman method is used to grow large single crystals^19^. Our study is applied to crystals grown by the vertical Bridgman method. Bridgman crystals are used as a source material for polycrystalline thin films^20^, and the analysis in our study describes the electro-optical characteristics of solar cell absorber source materials. We report band tail properties, Hall mobilities for electrons and holes, and minority carrier lifetimes. For the first time, we show the impact of band tails on charge carrier transport and dynamics. Band tails can reduce radiative open circuit voltage^21,22^ in As-doped^23^ and undoped^24^ CdTe, thus reducing implied voltage. Here, we show that band tails also impact charge carrier mobility and lifetimes, thus increasing recombination losses.

A comparison of high-resistivity and As-doped single crystals identifies changes in electro-optical characteristics due to As doping. We find not only orders-of-magnitude-higher electrical conductivity, which is expected for doped crystals, but also increased band tails, reduced mobilities, and reduced carrier lifetimes. These changes occur not due to grain boundaries, but rather due to bulk defects. The advanced single-crystal growth that is used in some samples in this study mitigates some of the losses. Results suggest that charge carrier scattering and recombination might occur not due to (largely unavoidable) point defects, but rather due to dopants or other aggregates.

## Results

### Defect and free carrier absorption below the bandgap

**Figure 1:**
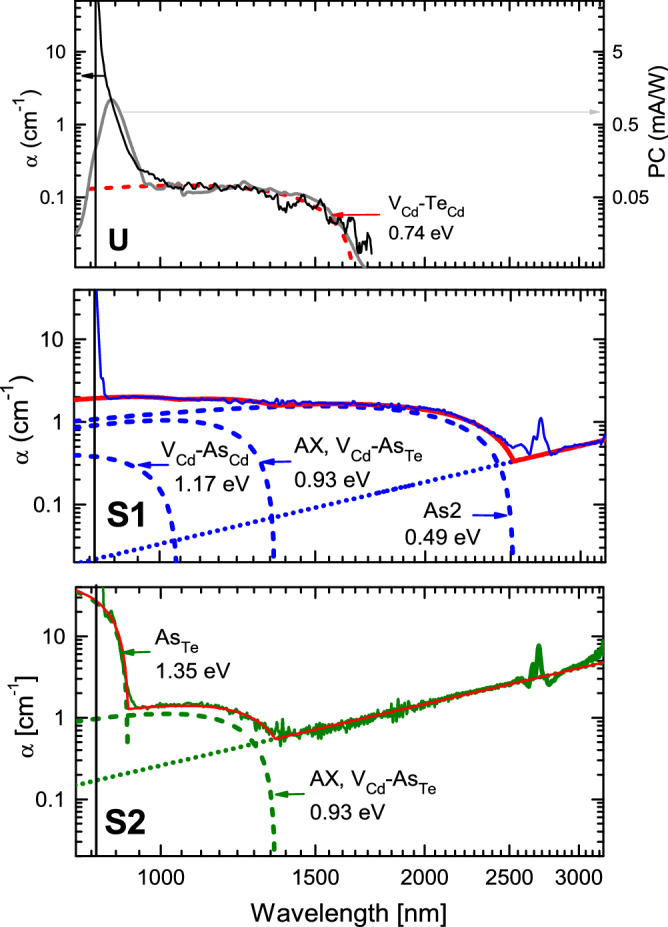
Absorption (left axis; black, blue, and green solid slides) and photoconductivity (right axis; grey solid line) spectra for undoped (U) and As-doped (S1, S2) single-crystal CdTe. E_g_ = 1.51 eV was assumed and is shown as a solid vertical line. Absorption coefficient error is 0.05 cm^−1^. Dashed lines approximate photoionization fits for traps, while dotted lines indicate free carrier absorption. Sharp peaks at ~ 2700 nm are measurement artifacts. Tentative peak assignments to electronic defect states are shown.

To analyze absorption below the bandgap, we used near infrared (NIR) transmission measurements with an integrating sphere. (Lock-in detection was used for transmission spectroscopy of undoped CdTe single crystals^25^. We find that simple instrumentation has sufficient sensitivity for the measurements reported here.) Absorption spectra for crystals: high resistivity CdTe (U), standard As-doped CdTe (S1), and accelerated crucible rotation grown As-doped CdTe (S2) are shown in Fig. 1. A solid vertical line indicates bandgap E_g_ ≈ 1.5 eV^24,26^, and for defect and band tail analysis, we consider absorption at < 1.5 eV. Doped samples S1 and S2 have higher optical absorption coefficients than the undoped sample U. This is attributed to increased band tails for group-V-doped CdTe^23^, differences in defect absorption (discussed below), and free carrier absorption in S1 and S2. All these features are important for CdTe optoelectronic devices. For example, band tails in polycrystalline CdTe have been observed using cathodoluminescence^23^, photothermal deflection spectroscopy (PDS)^24^, and absolute photoluminescence (PL)^27^. Our data shows that tails are also present for single-crystal CdTe, which indicates that grain boundaries and dopant accumulation at grain boundaries are not required for tail formation.

To confirm the band tail spectra for crystal U (where absorption is an order of magnitude lower than in doped crystals), we obtained the photoconductivity spectrum^28^; this data is also shown in Fig. 1. In comparison to absorption, the photoconduction spectrum has similar shape at > 900 nm due to the same defect absorption. Because the absorption coefficient is low at long wavelengths, the data reflects bulk defect absorption. However, photoconduction reduces at shorter wavelengths (< 900 nm) due to the impact of surface recombination on carrier lifetimes (see carrier lifetime analysis in “Charge carrier dynamics” section). Therefore, band tails in the single-crystal absorber bulk can be determined reliably using both optical (absorption) and electrical (photoconduction) measurements.

The data in Fig. 1 shows that the band tails have a non-Urbach (nonexponential) spectral shape, and it is important to consider their possible origin. In polycrystalline CdTe, similar nonexponential band tails were recently reported^24,27^. The sub-bandgap absorption coefficient of 100–1000 cm^−1^, determined in Ref.^24^ for polycrystalline CdTe deposited by radio frequency sputtering and close-space sublimation, is by order of magnitude larger, indicating a large concentration of defects. For large polycrystalline grains (> 10 µm) in passivated CdSeTe films, the absorption coefficient at 1000 nm was found to be ~ 0.1 cm^−1^, similar to the values for crystal U in Fig. 1. However, we also show that band tail absorption extends to longer wavelengths, which can limit radiative open circuit voltage in solar cells.

We now consider defect states that might contribute to band tails. As shown in Fig. 1, the spectra can be interpreted using free carrier absorption (dotted lines) and defect photoionization (dashed lines) spectra with threshold energies (numbers provided on the plot). (Such assignments are tentative because detailed defect models have not yet been developed, but identification of defect and free carrier absorption is important for material improvement.) First, dotted lines indicate free carrier absorption in doped samples S1 and S2. As expected for the free carrier density, which was determined using Hall measurements (see next section), free carrier absorption increases at longer wavelengths. Free carrier absorption is stronger in S2 due to higher doping. Such wavelength dependence *α* ∝ *λ*^2.3^ is typical for complex absorption by acoustic, optical phonons and impurities^29^. Second, dashed lines indicate defect photoionization spectra fits (several defects per sample). For defect photoionization spectra, we assume peak function spectral shapes, as described in Ref.^30^. These spectral shapes are discussed below. Finally, solid lines show the sum of defect photoionization fits and free carrier absorption.

The absorption spectrum for crystal U is simpler (Fig. 1). A single peak function with photoionization threshold at *E*_*TH*_ = 0.74 eV was used. This defect photoionization energy is consistent with published values for V_Cd_−Te_Cd_^31^. Different spectral features are present for crystals S1 and S2. Dopant As_Te_ photoionization threshold is tentatively shown at 1.35 eV^32^, but its contribution is small, consistent with low dopant activation. Using a *E*_*G*_ = 1.51 eV CdTe bandgap, As_Te_ activation energy is estimated to be *E*_*A*_ = 0.16 eV for S2 (here *E*_*A*_ = *E*_*G*_ − *E*_*TH*_). For S1, the peak with activation energy of 0.41 eV could be assigned to V_Cd_−As_Cd_. Transition energy for the AX center As_Te_−V_Cd_ is 500 meV, and this contribution seems to be present for both S1 and S2. Contributions from As_Te_ and the AX center are sufficient to fit the spectrum for S2. For S1, an additional spectral form with 0.49 eV threshold is needed. Using first principles, an As_2_ dimer defect was found at similar energy^13^.

In addition to the point defects summarized above, extended defects can also have optical signatures. For example, microscopic analysis identified As precipitates with composition As_6_CdTe (As_6_ clusters possible; size of 1–2 nm) or As_2_Te_3_^15^. Such defects with 234–284 meV activation energy and (0.37–4.14) × 10^−^^18^ cm^2^ capture cross section have been observed in polycrystalline CdTe films^33^. Thus, more complex complexes can also contribute to optical absorption. Regardless of the chemical identity of spectral features in Fig. 1, we find that doped samples S1 and S2 have more complex absorption spectra, leading to more complex carrier dynamics, consistent with the more complex defect states introduced by dopants. Mobility is also reduced due to the more abundant defects in doped crystals S1 and S2. Lifetime and mobility data are analyzed in the next sections.

### Temperature-dependent carrier density and mobility

**Figure 2:**
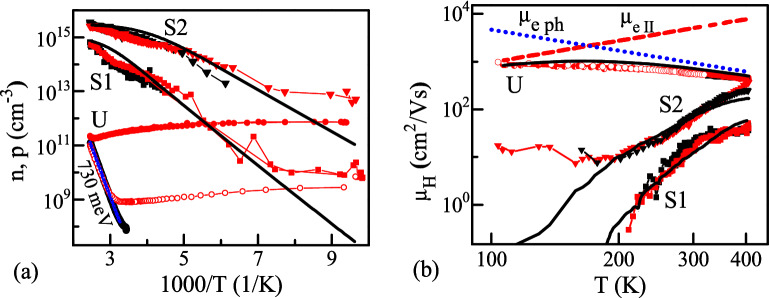
Temperature-dependent carrier density (**a**) and (photo)Hall mobility (**b**). Data are shown as circles for crystal U, as squares for crystal S1, and as triangles for crystal S2. Black symbols indicate measurements in the dark, while red symbols indicate photoconductivity (**a**) and photo-Hall (**b**) data measured using 850 nm excitation. For filled red symbols, excitation power density was 0.5 mW/cm^2^. For open red circles (sample U), excitation power was 0.6 *µ*W/cm^2^ in (**a**) and 60 µW/cm^2^ in (**b**). Solid lines in (**a**) are electron (U) and hole (S1, S2) density activation fits. Solid black lines in (**b**) are electron (U) and hole (S1 and S2) mobility fits. Dotted and dashed lines in (**b**) show phonon and ionized impurity scattering contributions to electron mobilities for crystal U.

Over the 100–400 K temperature range, sample U has low background (dark) carrier density, n < 10^12^ cm^−3^. Increased hole density (by 4–5 orders of magnitude) for S1 and S2 indicates p-type doping with As. Figure 2(b) shows Hall and photo-Hall measurement results. Using CW photoexcitation with 850 nm laser diode, enabled us to increase majority carrier density and measure photo-Hall mobility in U sample at low temperatures. In doped samples carrier density increase was low compared to equilibrium values due to high hole density and fast recombination (generated carrier density is proportional to carrier lifetime). We first discuss carrier density data in Fig. 2(a).

The modeling of carrier density temperature dependence (Fig. 2a) was used to estimate free carrier activation energy. For such modeling, we solved the charge neutrality equation^34^:

1 $$n + N_{A} - n_{A} = p + N_{D} - n_{D}$$

where *n* is the density of electrons in the conduction band, *p* is the density of holes in the valence band, $N_{A}$ is the total density of (built-in) acceptors, $N_{D}$ is the total density of donors, $n_{A}$ is the density of nonionized acceptors, and $n_{D}$ is the density of nonionized donors. All terms in Eq. (1) are temperature (*T*) dependent via the following expressions:

2 $$n = N_{C} \exp \left[ {\frac{{E_{F} }}{kT}} \right],\,\,\,p = N_{V} \exp \left[ { - \frac{{E_{F} + E_{G}}}{kT}} \right]$$

3 $$n_{D} = \frac{{N_{D} }}{{1 + g_{D}^{ - 1} \exp \left[ {\frac{{ - E_{D} - E_{F} }}{kT}} \right]}},\,\,n_{A} = \frac{{N_{A} }}{{1 + g_{A}^{ - 1} \exp \left[ {\frac{{E_{F} + E_{G} - E_{A} }}{kT}} \right]}}$$

where $E_{G}$ is the bandgap of the semiconductor, $E_{F}$ is the Fermi energy, *k* is the Boltzmann constant, $E_{A}$ and $E_{D}$ are acceptor and donor activation energies, and $g_{A}$ = 4 and $g_{D}$ = 2 are the degeneracy factors. Activation energies are considered absolute, with zero energy (reference) chosen at the conduction band edge. The valence band energy is negative. $N_{C}$ and $N_{V}$ are the temperature-dependent conduction and valence band densities of states, assumed to be $N_{V} = 1.27 \cdot 10^{25} \left( {T/300} \right)^{1.5}$ and $N_{C} = 9 \cdot 10^{24} \left( {T/300} \right)^{1.5}$, respectively, in units of ${\text{m}}^{ - 3}$.

The variable parameters *N*_*A*_ and *N*_*D*_, *E*_*D*_ and *E*_*A*_ were used to solve Eq. (1) with Wolfram Mathematica for the Fermi energy at different temperatures, which enabled to approximate experimental free carrier density *n*, *p* temperature dependences by least square method. These solutions for the majority carriers in U (electrons), S1 (holes), and S2 (holes) are given as solid lines in Fig. 2(a). The approximate analytical solution^35^ was used to confirm the numerical results.

The temperature dependence of the electron density in sample U provided activation energy *E*_*A*_ = 0.76 ± 0.05 eV. This value should correspond to the midgap deep acceptor, and we note that absorption at this energy was observed in Fig. 1. The defect assignment is not certain, but the Te antisite and Cd vacancy complex has an activation energy of 0.79 ± 0.06 eV (absorption is shown in Fig. 1)^27^. In p-type samples S1 and S2, the values for *p* = *N*_*A*_ − *N*_*D*_ were found to be 1 × 10^15^ cm^−3^ and 3 × 10^15^ cm^−3^, respectively. Activation energies were found to be *E*_*A*_ = 200 meV for S1 and *E*_*A*_ = 140 meV for S2. Activation energies *E*_*A*_ = 262–290 meV and *E*_*A*_ = 88–100 meV were determined from thermoelectric-effect spectroscopy (TEES) in similar As-doped CdTe single crystals, suggesting a common origin of such defects^14^. First principles analysis in Ref.^14^ identified these defects as AX centers and As_2_ dimers. From our data, the compensation for AX and As_2_ donors was found to be *N*_*D*_ = 2 × 10^15^ cm^−3^ and 1.2 × 10^16^ cm^−3^, respectively. Taken together, our results indicate that S2 exhibits more efficient doping.

Next, we consider the Hall and photo-Hall mobility data in Fig. 2(b). Electron mobility for compensated crystal U has weak temperature dependence and ranges from 1000 to 300 cm^2^/(Vs) at 100–300 K. Due to compensation, and hence the presence of ionized impurities, electron mobility is lower than for n-type single-crystal CdTe (10^4^ cm^2^/Vs at 100 K and 900 cm^2^/Vs at 300 K)^36^. To understand the mechanisms that reduce mobility in compensated CdTe single crystals, data for sample U was fit to phonon *µ*_*ph*_(*T*) ∝ *T*^−3/2^ and ionized impurity *µ*_*ii*_(*T*) ∝ *T*^3/2^ scattering terms^36^, which are additive according to the Matthiessen’s rule:

4 $${1}/ \mu\left( T \right) = {1}/ \mu_{ph} \left( T \right) + {1}/ \mu_ {ii} \left( T \right).$$

This fit is shown in Fig. 2(b) as a solid line, while phonon and ionized impurity scattering components are indicated by dotted and dashed curves, respectively. As this fit shows, room-temperature mobility is limited by ionized impurity scattering (*µ*_*ii*_(*T*) term).

We now consider hole mobility in doped crystals. Due to larger hole effective mass, hole mobility in CdTe is lower (about 100 cm^2^/(Vs))^37^. As indicated by temperature dependence in Fig. 2(b), hole mobility in As-doped samples is further reduced due to impurity scattering. Sample S2 has *µ*_*h*_ = 70 cm^2^/(Vs) at 300 K, and mobility is reduced to *µ*_*h*_ = 20 cm^2^/(Vs) for S1. Photo-Hall hole mobilities (shown as red symbols in Fig. 2(b)) are similar, which can be explained by AX center compensation. According to this mechanism, photogenerated electrons can be captured to AX centers and thus disappear from conduction, while photogenerated holes (partially) neutralize acceptors, increasing their ionization. As a result, optical excitation increases hole density and does not change carrier type (ionized impurity scattering is not reduced considerably, as excited carrier density is much lower than compensation in p-type samples).

The mobility drop at low temperatures in p-type crystals is much sharper than expected from ionized impurity scattering (Fig. 2(b); ionized impurity scattering for holes has similar temperature dependence as for electrons). Such strong temperature dependence can be explained by carrier localization in band tails and trap-controlled mobility^37^. The Urbach band tails are obscured by defect absorptions and thus are not clearly resolvable in Fig. 1. Using this Urbach model, we fit the band tail and trap-controlled mobility to a modified multitrapping equation^38^:

5 $$\mu_{U} (T) = \mu(T)/(1 + 2\alpha N_{L} /N_{V} \exp (x_{F} \alpha)/F_{ - 1/2} ),$$

where *α* = *kT/E*_*U*_; *E*_*U*_ is the Urbach energy; *F* are the Fermi integrals; *N*_*v*_ and *N*_*L*_ are the density of states in the valence band and localized states density, respectively; *x*_*F*_ = *E*_*F*_*/kT* is the dimensionless Fermi level; and *µ* (*T*) is the phonon and ionized impurity limited mobility for holes (Eq. 4). Levels *x*_*F*_ were calculated from hole density (*p*_0_) temperature dependencies in Fig. 2(a) using the Nilsson approximation^39^.

From fits of temperature-dependent mobilities for S1 and S2 in Fig. 2(b), we determined the densities of states in localized defect bands: *N*_*L*_ = 8 × 10^17^ cm^−3^ in S2 and 3 × 10^16^ cm^−3^ in S1. The larger *N*_*L*_ value for S2 can indicate a larger density of inactive As form (for example, (+ 2/ + 1) level of (As_i_As_Te_) at 0.94 eV from valence-band maximum (VBM) and (+ 1/0) level at 0.55 eV from VBM and (As_i_As_i_) complexes, with ionization levels at 0.58, 0.98, and 1.21 eV ^13^) forming band modulation. The smaller *N*_*L*_ value for traps in S1 can indicate large clusters of deep As_2_ defects^15^. Reduction of hole density at low temperatures indeed induces dominant hole localization in deeper Urbach tails; thus, the mobility drop at low T is much sharper than expected from only ionized impurity scattering. From temperature-dependent mobility fits to Eq. (5), we obtained Urbach energies *E*_*U*_ = 69 meV (S1) and *E*_*U*_ = 42 meV (S2). Therefore, sample 2 exhibits a shallower Urbach tail and larger mobility. At the lowest temperatures, when all holes are localized to Urbach tails, still considerable weakly T-dependent mobility in sample S2 is observed (10–20 cm^2^/(Vs)), which can be explained by hole transport close to the Fermi level in band tails or by not activated As defects due to high *N*_*L*_ density in S2. Homogeneously dispersed traps in S2 can have overlapping wave functions, which provides impurity conduction. While in S1, the precipitates can form drops that are not connected in space and thus do not form conduction, but still generate Urbach tails due to induced strain disorder. Crystal growth studies indicated a reduction in density of such microscopic defects when accelerated crucible rotation was used for S2 (but not S1).

### Charge carrier dynamics

**Figure 3:**
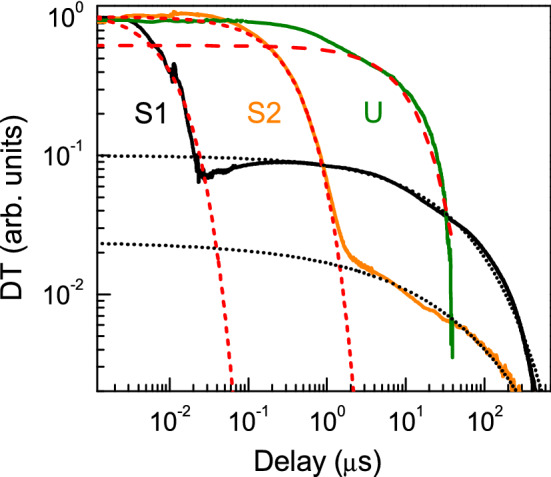
Transient absorption transients for crystals U, S1, and S2, spanning 1 ns to 1 ms timescale (Temperature T = 80 K, excitation fluence = 3 mJ/cm^2^). Dashed lines for all samples show exponential fits that describe Shockley–Read–Hall (SRH) recombination. Dotted lines for S1 and S2 indicate stretched exponent fits for decay tails.

**Figure 4:**
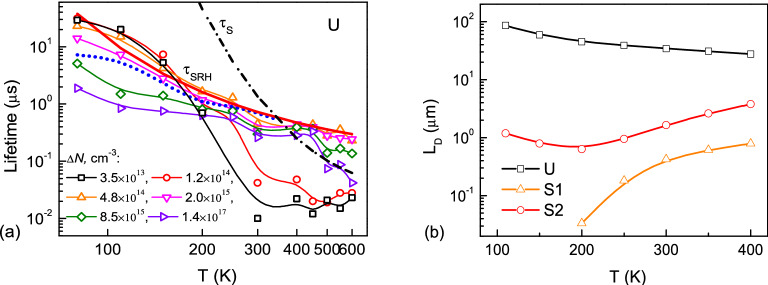
Charge carrier dynamics analysis for crystal U. (**a**) Symbols: temperature and injection dependent TA lifetimes. For comparison, the dotted blue line shows lifetimes from PL decay. The thick solid curve corresponds to SRH lifetime approximation. Surface lifetime at highest excitation is shown by the dash-dot line. (**b**) Temperature-dependent charge carrier diffusion length estimate from SRH lifetimes in (**a**) and mobility in Fig. 2(b).

As shown in Fig. 3, charge carrier dynamics occur on different timescales in undoped (U) and doped (S1, S2) crystals. This data was measured at 80 K. Transient absorption decays at other temperatures and at different excitation fluences are given in the Supporting Information (SI, Fig. 4S).

To fully describe the TA data (Fig. 3 and SI), we need to consider two distinct mechanisms. The first is Shockley–Read–Hall (SRH) recombination due to defect states in the crystal bulk and at the surface. Such dynamics are described as exponential decays. By varying the temperature and excitation fluence, we can separate bulk and surface recombination contributions, and differentiate radiative recombination at high excitation fluence. Such analysis is given below. For samples S1 and S2 with increased band tails, exponential decay models are not sufficient, and additional much slower (µs to ms) dynamics are observed. These slow dynamics can be described by stretched exponential functions, indicated as dotted lines in Fig. 3 (exponential fits are shown as dashed lines). Stretched exponential models describe hopping transport and tunneling recombination. For sample U, only the SRH recombination mechanism^40^ is dominant, whereas both SRH and hopping/tunneling are present in doped crystals with larger band tails. We first analyze SRH recombination and report temperature-dependent charge carrier lifetimes and diffusion length, and then consider dynamics in band tails of doped samples.

Representative TA decays with fits in a wide excitation and temperature range are shown in the SI (Fig. 4S). Higher temperature and low injection TA decays for crystal U are close to single exponential and correspond to SRH lifetime. At low temperatures and high fluences, we observed faster initial decay part due to radiative recombination impact. TA data was analyzed using a single exponential fitting model of initial decay parts, and lifetimes from this analysis are shown in Fig. 4 (the carrier densities in Fig. 4 were calculated for the decay initial parts^55^ according used excitation fluences at 0.1, 0.2, 0.4, 0.8, 1.6, 6.3 mJ/cm^2^). We found that lifetime in the U sample has strong temperature dependence, where *τ*_*fit*_ = 10 ns at 300 K and the lowest excitation fluence, and *τ*_*fit*_ = 30 µs at 80 K.

In addition, in Fig. 4(a) and in the SI (Fig. 3S), we show that time-resolved photoluminescence (TRPL) lifetimes (obtained using the same single exponential decay model) agree well with TA results.

We analyze decay rates using the following equation:

7 $$1/\tau = 1/\tau_{Rad} + \, 1/\tau_{S} + \, 1/\tau_{SRH}$$

where the radiative bimolecular rate is 1/*τ*_*Rad*_, surface recombination rate is 1/*τ*_*S*_, and nonradiative bulk recombination rate is 1/*τ*_*SRH*_*.* The radiative lifetime *τ*_*Rad*_ = 1/(*B* × *ΔN*^*2*^), where *B* is the bimolecular radiative coefficient. This *τ*_*Rad*_ had only impact at *ΔN* ≥ 2 × 10^15^ cm^−3^ and *T* < 200 K (see Fig. 4(a); note that *B* ~ *T*^−3/2^ temperature dependence is valid, as shown in the SI).

Recombination at the surfaces is typically described by surface lifetime *τ*_*S.*_ Its impact in sample U was fitted using the relation *τ*_*S*_ = *d/2S* for fundamental decay mode during surface recombination on both sample surfaces at stationary conditions, where the surface recombination velocity *S* relation (S1) from the SI was used. The increase in carrier density led to an increase in surface recombination velocity and a considerable reduction in lifetime at high temperatures (see Fig. 4(a); solid curves show $1/\tau_{{{\text{nonrad}}}} = 1/\tau_{S} + \, 1/\tau_{SRH}$ at different excitations). The *E*_*S*_ = 170 meV effective surface barrier was determined to be the same as from PL measurements (see details in the SI); a similar high injection *S*_0_ = 4 × 10^5^ cm/s value at 300 K was obtained. Calculations indicate that *S* increases from 5000 cm/s at 10^15^ cm^−3^ to 2.2 × 10^4^ cm/s at 1.4 × 10^17^ cm^−3^ and 300 K.

Finally, the obtained *τ*_*SRH*_ lifetime in sample U decreased strongly with temperature (from 30 µs to 0.3 µs; see red curve in Fig. 4(a)), which can be related to changing with temperature SRH trap capture parameters^41^. On the other hand, As-doped samples exhibited much shorter SRH lifetimes than the U sample (< 10 ns in S1 and < 1 µs in S2). This indicates that the p-type doping causes an increase in nonradiative SRH trap density. The prompt exponential lifetimes in doped samples were determined solely by fast SRH process. The surface and radiative decay impact was considered negligible due to much faster SRH decay (see the SI for details).

Finally, using SRH lifetimes (indicated by smoothed solid lines in Figs. 4 and Fig. 5S at 4.8 × 10^14^; 3.4 × 10^16^; 2 × 10^15^ cm^−3^ for U; S1; S2, respectively) and carrier mobilities (determined by photo-Hall effect; Fig. 2), we calculated temperature dependencies of diffusion length in the samples to evaluate the impact of As doping (*L*_*D*_ = (*τ* × *µkT*/*e*)^1/2^). It must be noted, that diffusion length and all carrier recombination parameters are carrier density dependent^42^, thus determination of electron and hole diffusion lengths is a complex and ambiguous task. As observed, As doping strongly reduces diffusion length due to lifetime drop in comparison to high-resistivity U sample (Fig. 4(b)). Advanced growth of the S2 sample provided much better diffusion length than the standard sample due to longer lifetime and greater mobility. Therefore, we found this growth method for nonradiative defect reduction to be effective for As-doped CdTe applications. Diffusion length slightly increases with temperature in As-doped samples, indicating that solar cell self-heating would not reduce their contact efficiency. In sample U, diffusion length strongly exceeds the typical thicknesses of active solar cell layers; thus, its temperature dependence should have minor impact.

### Decay tails

The decay tails can provide information on the presence of traps and band tails in As-doped samples. At carrier densities below 10^15^ cm^−3^ in the S2 sample and below 10^16^ cm^−3^ in the S1 sample, the lifetimes were strongly increased (i.e., decay tails appeared; see Fig. 3) compared to the SRH lifetime (see Fig. 5S in the SI). These threshold carrier densities can correspond approximately to present trap densities in the As-doped layers, as at higher excitations, decay tail amplitudes saturated (Fig. 4Sb and c). These densities (10^15^ cm^−3^ in S2 and 10^16^ cm^−3^ in S1) are similar to compensating donor densities determined by photo-Hall. Therefore, the tail decays can be explained by compensation in the p-doped samples and thus electrostatic bandgap fluctuation^43^. The decay tails were fitted with standard stretched exponential decay^40^ *DT* ~ exp(−(*t*/*τ*)^*β*^) relation (Fig. 3). In the S1 sample, the parameters of *τ* = 35 *µ*s and *β* = 0.5 were obtained. S2 had a five times weaker stretched decay amplitude due to a lower number of defects, and the parameters were smaller: *τ* = 19 *µ*s and *β* = 0.35. The latter indicates larger separation between the shallow localized tail states in S2. The temperature increase led to the suppression of decay tails due to activation of carriers from these tail (donor) states, as observed by DT in other compensated materials^44,45^.

## Discussion

Precise optical absorption spectroscopy allowed us to obtain sub-bandgap defect tail absorption in undoped and As-doped CdTe crystals. Different defects (As_Te_, V_Cd_−As_Cd_, V_Cd_−As_Te_, As_2_, V_Cd_−Te_Cd_) were identified. Time-resolved pump-probe with temporal resolution in the 80–600 K range provided bulk recombination lifetime in the CdTe material. Combining the lifetime data with photo-Hall mobility allowed us to calculate the diffusion length. In undoped CdTe, extraordinarily long lifetimes (30 µs) at low temperatures were determined and explained by the low concentration of nonradiative defects. The bimolecular recombination coefficient with *T*^*−*3/2^ temperature dependence and surface recombination rate activated with 170 meV effective surface barrier were weakly impacting this slow lifetime. As doping provided reduction of lifetime by a few orders of magnitude. Nevertheless, the enhanced crucible rotation growth allowed us to improve SRH lifetime, mobility, and carrier activation by an order of magnitude in comparison to the standard sample. That improvement allowed for a fivefold increase in its diffusion length. The observed slow stretched exponent decay tails in As-doped samples were explained by electrostatic bandgap fluctuations arising due to compensation.

## Methods

### Crystal growth and electrical measurements

Single-crystal high-resistivity n-type CdTe (sample U) was grown at Washington State University using the vertical Bridgman method^5,14^. Dislocation densities for similar samples were (2–5) × 10^4^ cm^−2^. For optical measurements of samples without As, we selected an In (≈ 3 × 10^17^ cm^−3^) and Er (1.5 × 10^17^ cm^−3^) co-doped high-resistivity 0.5 mm-thick crystal, where about 10^17^ shallow donor concentration leads to the largest resistivity^46^. It is believed that the compensation arises due to the simultaneous impact of shallow indium donor at *E*_*C*_ − 14 meV^47^ and *E*_*V*_ + 120–150 meV acceptor^48^ and two intrinsic deep defects (Te antisite and the Cd vacancy in different charge states)^49,50^. Co-doping with Er allows for a deep level at 0.8 eV that removes the V_*Cd*_^2−^type complexes with Te antisite or their complexes. Er provides shallow acceptor level at 72 meV^51^.

The As-doped sample CG166 (sample S1) was grown by standard method, while the second As-doped sample CG203 (sample S2) was grown using accelerated crucible rotation crystal growth^52^, which improved crystal quality and As activation.

Sample U was grown under Te-rich condition and arsenic doped crystals S1 and S2 were grown under excess Cd condition, by simply adding elemental Cd to the pre-compounded CdTe (99.99995% purity) from 5 N Plus Inc. The amount of excess Cd including from the dopant Cd_3_As_2_ is 7 × 10^18^ cm^−3^ for S1 and 3.7 × 10^19^ cm^−3^ for S2. These numbers are greater than the maximum solid solubility of Cd in CdTe ~ 5 × 10^18^ cm^−3^^53^, and the grown crystals are expected to be Cd-rich, with precipitates and other secondary phases of Cd including with dopants. Undoped and phosphorus doped crystals grown similarly with excess Cd are reported to show such phases^19,54^.

XRD patterns of the samples were measured using Rigaku SmartLab diffractometer equipped with 9 kW rotating Cu anode X-ray tube and linear detector (D/Tex) in the range of 10° ⩽ 2θ ⩽ 90° working in Bragg–Brentano focusing geometry. The patterns are provided in SI together with energy-dispersive X-ray spectroscopy (EDX) and glow discharge mass spectrometry (GDMS) results, proving samples are Cd-rich and with low concentration of impurities.

For the high-resistivity (U) long-bar shape sample (0.5 mm thick), electrical measurements were performed using Ohmic contacts prepared by soldering with indium. For As-doped p-type samples, Ohmic contacts were prepared by physical vapor deposition of PbTe doped with BaF_2_. Van der Pauw geometry samples were 2.2 mm and 1.9 mm thick, respectively. Contact islands were covered with Au on top, and samples were annealed at 400 °C for 2 min. Increase of hole density with temperature revealed As acceptors.

Carrier density and mobility were investigated by Hall and photo-Hall techniques. The experimental setup consisted of a magnet (with 1 Tesla magnetic induction), liquid nitrogen-cooled cryostat, Keithley 6430 source meter, and Keithley 6514 electrometer for Hall voltage registration. For photo-Hall measurements, excitation with an 850-nm light-emitting diode (LED) was used. LED intensity was regulated by current or neutral density filters. Photoconductivity spectra were measured using the Keithley 2401 source meter and tunable optical excitation provided by a femtosecond optical parametric amplifier (Orpheus, Light Conversion).

### Optical spectroscopy

Absorption spectra were measured with a Perkin Elmer Lambda 950 UV-NIR absorption spectrometer with integrating sphere. Transmission spectra T were corrected by subtracting sample reflection R to get absorption spectra A (A = T – R). The low photometric noise of the spectrometer (0.0001 optical density units) allowed us to measure optical density down to ≈ 0.001, resulting in absorption coefficient error ≈ 0.1 cm^−1^ for 0.5 mm sample thickness. The low light scattering from the polished single-crystal samples and the use of the integrating sphere enabled such optical sensitivity. Spectra were smoothed using a 10-point running average to reduce noise.

Recombination dynamics and charge carrier lifetimes were investigated with time-resolved pump-probe and photoluminescence (TRPL) techniques. The 1053-nm excitation wavelength was used for two photon carrier generation (CdTe bandgap ≈ 1.5 eV), which allows reliable recombination analysis in the crystal bulk ^55^. Excitation optical path was equal to sample thickness with almost homogeneous carrier in-depth distribution. The probing was performed at 20 deg angle with respect to pumping beam (probe diameter was set three times smaller than the pump diameter). Excitation intensity was varied using rotating half-wave plate before a Glan polarizing prism. For pump-probe measurements, a 1550 nm CW probe (Eblana Photonics), 5-GHz-bandwidth InGaAs detector, and 6-GHz oscilloscope setup were used^56^. For detection of TRPL decays and PL spectra at room temperature, a Hamamatsu streak camera was used in the integrating mode. For temperature-dependent TRPL measurements, an electrooptic detector was used with a MS520i spectrograph and an intensified Dhyana CMOS camera^57^. PL was collected from whole sample thickness using 10 cm lens. Temperature was varied by placing the sample in CTI-Cryogenics nitrogen cryostat.

## Supplementary Information

Supplementary Information.

## Data Availability

All experimental data that support the findings of this study are available from the corresponding author upon reasonable request.
