# Supplementary material for: Impact of dopant-induced band tails on optical spectra, charge carrier transport, and dynamics in single-crystal CdTe
Source: Sci Rep. 2022 Jul 27;12:12851. doi: 10.1038/s41598-022-16994-7 (PMC9329450; doi:10.1038/s41598-022-16994-7)
Supplement: Supplementary file 1 — Supplementary Information. [file 41598_2022_16994_MOESM1_ESM.docx]

**Supplementary information**

**Impact of dopant-induced band tails on optical spectra, charge carrier transport, and dynamics in single-crystal CdTe**

Patrik Ščajev^1*^, Algirdas Mekys^1^, Liudvikas Subačius^2^, Sandra Stanionytė^2^, Darius Kuciauskas^3^, Kelvin G. Lynn^4^, Santosh K. Swain^4^

^1^ Institute of Photonics and Nanotechnology, Faculty of Physics, Vilnius University, Saulėtekio Ave. 3, LT-10257, Vilnius, Lithuania

^2^ Optoelectronics Department, Center for Physical Sciences and Technology, Saulėtekio Ave. 3, LT-10257, Vilnius, Lithuania

^3^ National Renewable Energy Laboratory, 15013 Denver West Parkway, Golden, Colorado 80401, USA

^4^ Center for Materials Research, Washington State University, Pullman, Washington 99164, USA

* Patrik.scajev@ff.vu.lt

**Photoluminescence spectra and decays**

Photoluminescence spectra shown in Fig. 1S were measured using 1053 nm (below bandgap) excitation are red-shifted (in comparison to one-photon excitation photoluminescence emission) because of the reabsorption for higher energy photons [1,2]. This reabsorption more strongly affects two-photon excitation data, when carriers can be photogenerated at a significant distance from the crystal surface.

Undoped sample exhibits narrowest spectrum due to dominant electron-hole recombination transitions. S2 has the strongest redshift due to electron to shallow acceptor trap luminescence at lowest excitations. Peak is broadened due to overlap of band-to-band and subbandgap electron to neutralized As_Te_ transitions [3]. At high excitations acceptors saturate and S2 peak restores to bandgap emission (other samples exhibit much lower shift with excitation).

**Fig. 1s.** Excitation fluence dependent photoluminescence spectra and decays for U (a,b), S1 (c,d) and S2 (e,f) samples at 300 K. Numbers in the legends indicate excitation fluence.

Next, we discuss PL decays. The PL timescale is hundreds of ns for sample U, tens of ns for S2, and less than 10 ns for S1. Decays are close to single exponential for S1 (lifetime 3-4 ns) but more complex for U and S2. To compare lifetimes for complex kinetics, we use analysis in Figure 2S. In Fig. 2S (a), we plot peak PL amplitude vs. excitation intensity. Similar high excitation peak PL intensity in all samples (*PL*(*t* =0)~ *B*Δ*N*(*t* =0)^2^; excited carrier density Δ*N* is proportional to excitation squared) indicates radiative lifetime is similar in all samples. In S2 integrated intensity is slightly lower, which can be partially a consequence of hole trapping to shallow As_Te_ acceptors. The data for all three samples can be described by a power law with the exponent of 3.6 ± 0.1. In agreement with these results, in high injection and two photon excitation a slope close to 4 is expected.

**Fig. 2S.** Excitation dependence of free carrier 850 nm PL peak intensity (a) and PL decay time in different delay times after excitation Δ*t* (b) at 2P excitation. Upper scale shows excited carrier density in cm^-3^. Dashed lines in (b) show surface lifetime approximations by (S1) equation in initial decay parts.

Lifetimes in Fig. 2S(b) were obtained by using exponential fitting at three time windows for U and S2 samples. In this analysis we also account for the surface recombination using simplified phenomenological relation (S1).

*S*(Δ*N*,*T*) = *S*_0_(1 + *N*_1_exp(*E_S_*/*kT*)/(Δ*N*+*N*_2_))^-1^×(*T*/300K)^1/2^ (S1)

Here *S* is the measured surface recombination velocity, *S*_0_ is the saturated surface recombination velocity at high injection, Δ*N* is injected carrier density, *k* is the Boltzmann’s constant, *E_S_* is the effective surface barrier. Factor (*T*/300K)^1/2^ accounts for carrier thermal speed temperature dependence. Analyzing theoretical *S* equations found in [4,5] it can be found that our equation should account for surface band bending, surface trap capture cross section, thermal activation energy and trap position in the bandgap (the three activation energies simplify to effective *E_S_*); as well as density of states, doping and thermal velocity. Our (S1) equation greatly simplified analysis as precise equation contains numerous parameters of unknown surface traps in CdTe.

For samples U and S2 *S*_0_ = 6x10^5^ cm/s and *N*_1_exp(*E_S_*/*kT*) = 3x10^18^ cm^-3^ were calculated using fits in Fig. 4a. *N*_2_ value for U was 4x10^16^ cm^-3^, while for S2 *N*_2_ = 10^17^ cm^-3^. Due to weak impact of surface recombination, in sample S1 lifetime is weakly dependent on excitation. At the highest excitations the *S* value saturates to constant value according to this model. In comparison, previously we determined similar *S* = 6x10^5^ cm/s for undoped CdTe at high injections (saturated at 10^19-20^ cm^-3^) using LITG method [6].

**Temperature dependence of PL lifetimes**

To learn more about the recombination mechanisms, we studied temperature-dependent PL lifetimes shown in Fig. 3S. From such data it is possible to distinguish radiative and SRH recombination because these processes have different temperature dependence. Data for the undoped sample U in Fig. 3S(a) shows that lifetimes increase between 50 K - 200 K and decrease at higher temperatures. The slope = 1.5 in the log-log graph at 50 - 200 K confirms that radiative (bimolecular) recombination dominates in this temperature range. This trend is shown as a dotted line in Fig. 3S (a). Radiative lifetime *τ_RAD_* = 100 ns at 300 K provides *B* coefficient 7×10^-11^ cm^3^/s according to relation *τ_RAD_* = 1/(*B*Δ*N*). This value is in good agreement with earlier studies [6]. We also observed (not shown) that PL decay peak intensity on temperature reduced with the similar slope, confirming *PL* ~ *B*(*T*)Δ*N*^2^ relation.

At temperatures 200 K to 600 K lifetimes decrease, which is attributed to the SRH recombination rate increase. Recombination rate increase between 300 – 600 K is larger than expected for SRH recombination due to the midgap states. Data is consistent with the activated process, such as due to the surface traps analyzed in the earlier section (Eq. S1). The dashed line in Fig. 3S (a) uses the model by equation (S1), providing *S*(10^17^cm^-3^) = 2.6×10^4^ cm/s at RT, *N*_1_ = 4×10^15^ cm^-3^ and *E_S_* = 170 meV effective surface barrier.

Surface recombination after two photon excitation occurs due to carrier generation in the bulk and drift/diffusion to the surface. It was also possible to measure surface recombination velocity directly, by using one photon excitation at 600 nm (absorption coefficient *α* = 5x10^4^ cm^-1^) resulting in ~10^19^ cm^-3^ initial carrier density near the surface. Values of *S* = 7×10^4^, 1.7×10^5^ and 3×10^5^ cm/s were calculated from initial decay parts (*τ_surf_* = 0.26, 0.12, 0.06 ns decay times) in S1, S2 and U samples, respectively. Relation *τ_surf_* = 1/(*αS*) was used as carriers were generated close to the surface and reabsorption was minimal. Latter values are close to estimated *S*_0_ values in **Photoluminescence spectra and decays** section.

**Fig. 3s.** Prompt PL lifetime temperature dependences in undoped sample (a) and delayed PL lifetime comparison in different samples (b). In (a) dotted line shows bimolecular, while dashed – surface recombination impact to the total recombination lifetime (shown by solid line) at 10^17^ cm^-3^ carrier density. Sample S1 exhibits fast and weakly temperature dependent decay due to different origin of recombination traps (small capture barrier). Straight lines show linear fits in the log-log plots.

We also observed delayed PL decay lifetimes, as shown in Fig. 3S b. This data indicates the impact of defects with different origin. They were studied by DT technique which is less sensitive to the surface traps as signal comes from full sample thickness.

**DT decay initial parts**

The representative decays of differential transmission are shown in Fig. 4S. They represent initial part of the decay dependence on excitation at different temperatures. In U sample high excitation decays become faster at *T* = 80 K due to bimolecular recombination. While in S1, S2 samples faster initial decay part emerges due to saturation of band tail states. The decay tail amplitude shows tendency of saturation at highest excitation due to limited amount of band tail states. Fig 5S provides excitation dependent lifetime data for doped samples, revealing the same band tail state saturation effect by lifetime reduction with excitation.

**Fig. 4S.** Representative initial DT decays at different excitations at 80 K, 300 K and 550 K in U (a), S1 (b) and S2 (c) samples. Numbers in the legends indicate excitation fluences in mJ/cm^2^. Slow decay tails in S1 and S2 samples are due to recharged trap states. The straight lines are exponential initial decay time fits at corresponding initial carrier densities as depicted in Fig. 5S. Note, that legend in Fig. 4S is in mJ/cm^2^, which corresponds to laser excitation fluences, while legend in Fig. 4 is in cm^-3^, where cm^-3^ corresponds to initial carrier density in the decay initial part (calculated according equations in ref [6]) – the carrier density reduces with time by few orders of magnitude and decay times in decay tails correspond to lower carrier densities.

**Fig. 5S.** Temperature dependences of prompt DT lifetimes at different injections in S1, S2 samples. Excited carrier density varies in 10^15^ – 10^17^ cm^-3^ range. For comparison, dotted blue lines show lifetimes from PL decay at 10^17^ cm^-3^. Thick solid curves correspond to free carrier SRH lifetime guide to eye approximations.

**Structural characterization**

Figure 6S shows the XRD patterns obtained from the CdTe grown using the vertical Bridgman method. XRD patterns were measured from a portions of the single crystals ground into a powder, where all peaks can be assigned to a zinc blende structure of CdTe (PDF# 01-070-8042) that contains no significant impurity structure. As incorporation does not change the lattice constant. No diffraction peaks of Te or Cd inclusions [7] were observed.

**Fig. 6S.** Powder XRD patterns of investigated samples.

EDX results show small excess of Cd. For comparison EDX characterisation in Cd rich CdTe single crystals provided 50.32% Cd and 49.68 Te [8].

We also report the chemical purity of the As-doped crystals (Fig. 7S), measured by glow discharge mass spectrometry (GDMS), with uncertainty 0.5 to 2 times the value for all the impurities, except C, N and O which has 5-fold uncertainty.

**Fig. 7S.** Impurity concentrations in As-doped crystals.

**References**

[1] X.-H. Zhao, M. J. DiNezza, S. Liu, C. M. Campbell, Y. Zhao, and Y.-H. Zhang, Appl. Phys. Lett, 105, 252101 (2014).

[2] D. Kuciauskas, A. Kanevce, J. M. Burst, J. N. Duenow, R. Dhere, D. S. Albin, D. H. Levi, and R. K. Ahrenkiel, IEEE J. Photovoltaics 3, 1319 (2013).

[3] G. Kartopu, O. Oklobia, D. Turkay, D.R. Diercks, B.P. Gorman, V. Barrioz, S. Campbell, J.D. Major, M.K. Al Turkestani, S. Yerci, T.M. Barnes, N.S. Beattie, G. Zoppi, S. Jones, and S.J.C. Irvine, Sol. Energy Mater. Sol. Cells 194, 259 (2019).

[4] S. W. Glunz, A. B. Sproul, W. Warta, and W. Wettling, J. Appl. Phys. 75, 1611 (1994).

[5] A. G. Aberle, S. Glunz and W. Warta, J. Appl. Phys. 71, 4422 (1992).

[6] P. Ščajev, S. Miasojedovas, A. Mekys, D. Kuciauskas, K.G. Lynn, S.K. Swain, and K. Jarašiūnas, J. Appl. Phys. 123, 025704 (2018).

[7] I. M. Dharmadasa, O. K. Echendu, F. Fauzi, N. A. Abdul-Manaf, O. I. Olusola, H. I. Salim, M. L. Madugu and A. A. Ojo, J. Mater. Sci.: Mater Electron 28, 2343 (2017).

[8] A. Nagaoka, K. Nishioka, K. Yoshino, D. Kuciauskas, and M. A. Scarpulla, J. Electron. Mater. 49, 6971 (2020).

**Acknowledgments**

The research was supported by the Research Council of Lithuania under the project No. S-MIP-19-34. This work was authored in part by the National Renewable Energy Laboratory, operated by Alliance for Sustainable Energy, LLC, for the U.S. Department of Energy under Contract No. DE-AC36-08GO28308. This material is based upon work supported by the U.S. Department of Energy’s Office of Energy Efficiency and Renewable Energy (EERE) under the Solar Energy Technology Office Award Numbers 38525. The views expressed herein do not necessarily represent the views of the U.S. Department of Energy or the United States government. The U.S. government retains and the publisher, by accepting the article for publication, acknowledges that the U.S. government retains a nonexclusive, paid-up, irrevocable, worldwide license to publish or reproduce the published form of this work, or allow others to do so, for U.S. government purposes.
